# Excess mortality in Belarus during the COVID-19 pandemic as the case study of a country with limited non-pharmaceutical interventions and limited reporting

**DOI:** 10.1038/s41598-022-09345-z

**Published:** 2022-03-31

**Authors:** Alexander Kirpich, Aleksandr Shishkin, Thomas A. Weppelmann, Alexander Perez Tchernov, Pavel Skums, Yuriy Gankin

**Affiliations:** 1 Department of Population Health Sciences, School of Public Health, Georgia State University, Atlanta, GA USA; 2 Department of Internal Medicine, University of South Florida, Tampa, FL USA; 3 Faculty of Mechanics and Mathematics, Belarusian State University, Minsk, Belarus; 4 Department of Computer Science, Georgia State University, Atlanta, GA USA; 5 Quantori, Cambridge, MA USA

**Keywords:** Health care, Diseases, Infectious diseases, Respiratory tract diseases, Epidemiology

## Abstract

Public health intervention to contain the ongoing COVID-19 pandemic significantly differed by country since the SARS-CoV-2 spread varied regionally in time and in scale. Since vaccinations were not available until the end of 2020 non-pharmaceutical interventions (NPIs) remained the only strategies to mitigate the pandemic spread at that time. Belarus in Europe is one of a few countries with a high Human Development Index where no lockdowns have ever been implemented and only limited NPIs have taken place for a period of time. Therefore, the Belarusian case was evaluated and compared in terms of the mortality burden. Since the COVID-19 mortality was low, the excess *overall* mortality was studied for Belarus. Since no overall mortality data have been reported past June 2020 the analysis was complemented by the study of Google Trends funeral-related search queries up until August 2021. Depending on the model, the Belarusian mortality for June of 2020 was 29 to 39% higher than otherwise expected with the corresponding estimated excess death was from 2953 to 3690 while the reported COVID-19 mortality for June 2020 was only 157 cases. The Belarusian excess mortality for June 2020 was higher than for all neighboring countries with an excess of 5% for Poland, 5% for Ukraine, 8% for Russia, 11% for Lithuania and 11% for Latvia. The relationship between Google Trends and mortality time series was studied using Granger’s test and the results were statistically significant. The results for Google Trends searches did vary by key phrase with the largest excess of 138% for April 2020 and 148% for September 2020 was observed for a key phrase “coffin”, while the largest excess of 218% for January 2021 was observed for “funeral services”. In summary, there are indications of the excess overall mortality in Belarus, which is larger than the reported COVID-19-related mortality.

## Introduction

The novel coronavirus SARS-CoV-2 was originally detected in December 2019 in Wuhan, China^1–5^ and within just three months^6^ rapidly spread around the world. The corresponding public health interventions to curb the pandemic, however, significantly differed between regions^7^. This happened since the spread of SARS-CoV-2 varied in time and scale as well as the overall preparedness of healthcare systems and hospitals that could be utilized simultaneously^8^. The large-scale vaccination interventions were not available until the end of 2020 due to time required for vaccine development and licensing^9–17^. As a result mandatory non-pharmaceutical interventions (NPIs) remained the only strategies to control the pandemic. The NPIs included multiple measures ranging from basic precautions and some social distancing to tracing of all infected individuals together with their contacts and complete lockdowns of non-essential public services and business activities.

Belarus in Eastern Europe is one of a few countries with a high Human Development Index (vhHDI)^18^ where no lockdowns have ever been implemented and only limited NPIs took place^7^. The available NPIs included some contact tracing, 14-days self-isolation for close contacts of laboratory-confirmed cases and for those returning from abroad, delay in class starting times for schools, remote learning for some universities, and increased frequency of public transportation to avoid crowding^19^. Since the social distancing measures were not strictly implemented or enforced, it remained up to individuals whether and how to change their behavior patterns^20^. In particular, the mask regimen was recommended but not evaluated for compliance^21^. Businesses and public services which required personal interactions, such as restaurants, gyms, barber shops, taxis, and theaters always remained fully operational. The mask regimen was not mandatory until November of 2020 and no country exit and entry limitations were implemented by Belarusian authorities until December of 2020. Even when the vaccination campaign eventually started in the very end of 2020^22^, the vaccination rates remained one of the lowest in Europe for a period of time^23,24^. As a consequence, Belarusians remained relatively free in their choices and in personal risks that they were willing to undergo up until the end of 2020. Even then, the newly mandated NPIs were mild in comparison to neighboring Poland, Russia, Ukraine, Lithuania and Latvia.

Given such circumstances, the thorough study of the Belarusian approach towards battling the COVID-19 pandemic is crucial, since the Belarusian cases illustrates: (1) the implications of limited NPIs in the country with high HDI and the universal healthcare system and (2) the limited reporting and limited publicly available surveillance data. In the meantime, despite aforesaid reporting such study is still possible since Belarus has a surveillance system comparable to the other countries with high HDI.

At first glance the lab confirmed COVID-19 mortality serves as a direct and unbiased measure of the epidemic burden since (almost) all such deaths are recorded properly. This measure, however, has flaws. In reality, most of the asymptomatic individuals and some individuals with mild (and even severe) symptoms remain untested. As a consequence, death of *untested* individuals is not reflected in COVID-19-induced mortality statistics. This also happens due to limited testing capacities which vary by region and over time. On the other hand, some *tested positive* deceased individuals may have had causes of death other than COVID-19. Those may include various pre-existing health complicating conditions such as deadly chronic diseases or recent severe traumas. For such individuals the exact cause of death is ambiguous but their deaths can be included in COVID-19 mortality.

To address this uncertainty in COVID-19 mortality the study of the *overall and excess mortalities* during the pandemic period comes to places. More specifically, the estimated change in the overall population mortality from *all causes* during the pandemic period provides a well defined measurement estimate of the epidemic toll for a given region which can be compared across regions^25,26^.

Such statistical estimates are robust since they (1) do not depend on the preformed amount of laboratory tests and (2) do not rely on proper identification of causes of death and related ambiguity. Belarus as a country does not participate in various regional monitoring initiatives such as a European mortality monitoring activity (EuroMOMO)^27^. Therefore, this work fills this knowledge gap for Belarus by studying the excess *overall* mortality and related characteristics such as the related Google Trends search queries^28^ during the epidemic period. This also complements the limited available knowledge about the Belarusian COVID-19 burden^25,29^. The limitations of such an excess mortality approach are that the exact (clinical) causes of each death are not reported for Belarus. Therefore, the excess mortality summaries by clinical cause cannot be directly attributed to COVID-19 as well as compared with such summaries for the neighboring countries which do report such data. They can only be interpreted as the overall “mortality burden” during the pandemic with potentially multiple underlying causes.

This work contains: (1) the analysis of officially reported overall death counts and excess mortality, (2) the demographic characteristic summaries and their incorporation into models, (3) the comparisons of mortalities via standardized scores with neighboring counties, (4) the studies of the auxiliary Google Trends search data and their relationship with mortality data during the pandemic period.

## Data sources

### Demographics and overall mortality

No individual level data have been used in this work. The analysis was performed on publicly available aggregated population summaries which did not include any individual level personal information. The annual population structure and annual mortality were downloaded from the National Statistical Committee of the Republic of Belarus (NSCRB)^30,31^. For the past ten years the NSCBR published population data *annually* as “Demographic Yearbook of the Republic of Belarus” and (as of November 2021) the latest available yearbook was published for 2019^32^. Therefore, for year 2020 the Belarusian census summaries from October 2019 were used instead.

The Belarusian *monthly* mortality from January 2011 to June 2020 were downloaded from the United Nations Demographic Statistics Database^33^. At authors knowledge as of November 2021 this is the most detailed and the most up to date overall mortality dataset^25^. In particular, the most recent edition “Belarus in Numbers 2021” by the NSCBR was released on April 29, 2021^34^ and *did not* include the annual mortality counts while they were included in pre-COVID-19 “Belarus in Numbers 2020”^35^.

### COVID-19 cases and mortality

The COVID-19 epidemiological data were reported by the Ministry of Public Health of the Republic of Belarus^36^. Since the beginning of the pandemic (March 2020) the reports were in the format of online press releases^36–38^ and daily numbers of confirmed cases, deaths and conducted tests. The daily updates were reported via the Ministry of Public Health official Telegram Messenger channel^38^. Since Telegram channel data were in unstructured text format the news agency onliner.by generated interactive visual summaries for incidence, recovery, and death counts^39^.

### Google trends

Google Trends *monthly* searchers^28^ from January 2015 until August 2021 were considered as auxiliary data to complement the existing knowledge. The following search query time series were considered: (1) “гpоб” (“grob”) i.e. coffin, (2) “поминки” (”pominki”) i.e. memorial service and (3) “pитуальныe уcлуги” (“ritualnie uslugi”) i.e. funeral services.

## Methods

### Excess mortality quantification

The study is focused on a retrospective analysis and comparison of the (standardized) Belarusian excess mortality with the neighboring countries. Broadly, the *excess mortality* during a period of time (e.g. month) is defined as the actual death counts minus the death counts that would have “normally expected”^26^, where “normally expected” can be interpreted pretty broadly. In this work the formal definition of “excess death” during a certain period (i.e. month) is the difference between the actual death counts minus the “expected counts” which are predicted from statistical modeling. Such modeling can incorporate auxiliary data such as population age characteristics over time. The approach is to formulate and to fit models to historic mortality data over *n* time periods (i.e. months) that directly precede the epidemic start date. Then the same models are used to produce the “expected” predicted mortality during the epidemic period for comparisons with the reported mortality data. The differences between reported and predicted produce the formal quantification of “excess mortality”.

### Standardized mortality scores

While the modeling efforts do provide a formal quantification of the “excess” mortality numbers they are lacking a meaningful way to compare those counts between countries or regions. The later comparison may be very much desired to compare the effectiveness of the applied NPIs.

The formal comparisons between countries (or regions) can be done via the *standardized scores*^40^. In particular, the *P*-scores allow to compare mortality counts for different time periods and populations of different regions with different sizes^41^. The scores are defined either non-parametrically or parametrically^26^ and both are evaluated in this work. The non-parametric version for *I* studied periods has the form:

1 $$\begin{aligned} P(t_i) = \frac{x(t_i) - \bar{x}(t_i)}{\bar{x}(t_i)}\, \text{ for } \, i=1,2, \dots , I, \end{aligned}$$

where $\bar{x}(t_i)$ is the average mortality of all $t_i$ periods from the past *n* years that precede the studied epidemic period.

The parametric version for *I* study periods has the form:

2 $$\begin{aligned} \mathscr {P}(t_i) = \frac{x(t_i) - \hat{\mu }(t_i)}{\hat{\mu }(t_i)}\, \text{ for } \, i=1,2, \dots , I \end{aligned}$$

where $\hat{\mu }(t_i)$ is the model-predicted population mortality for $t_i$ studied period based on the model fit using *n* years of data that precede the studied epidemic period. The differences between non-parametric (1) and parametric (2) and expected to be minor^26^. The scores $P(t_i)$ and $\mathscr {P}(t_i)$ are typically converted to percent for easier interpretation. For example, the converted score value of 120.47 indicates that mortality is exceeded by 120.47%. Since the mortality scores are on the same scale regardless of the studied population size, they can be easily interpreted and compared *between regions*.

The non-parametric (1) and parametric (2) scores are not limited to mortality data and corresponding time series. The same scores can be computed and studied for any time series such as Google Trends search queries which is also done in this work.

### Predictive models for parametric estimates

The model which is chosen to produce the expected estimates $\hat{\mu }(t_i)$ for $i=1,2, \dots , I$ for formula (2) has to account for (competing) processes which are simultaneously happening within the studied population and has to incorporate the ability to disentangle the effects of each process on the mortality. In particular, the model should at least incorporate annual seasonality of mortality^42^, potential time lags as well as changes in the studied population structure and size^43^. For COVID-19 the demographic change is crucial, since the Belarusian population is ageing and the number of high risk individuals older than 65 is increasing^44^. Such demographic features have not been incorporated in excess mortality studies earlier^25^, while such changes can potentially affect the mortality dynamics. Two time series models have been used: the Prophet forecasting model^45^ and an autoregressive integrated moving average (ARIMA) model^46^.

The Prophet model^45^ is implemented in R package prophet and consists of three main components: trend, seasonality and holidays. The model is formulated as:

3 $$\begin{aligned} y(t) = g(t) + s(t) + h(t) + \epsilon _t \end{aligned}$$

for a given time interval *t*. The formula (3) consists of dependent (response) model variable *y*(*t*) which represents mortality counts or search trend values, independent (exploratory) variables *g*(*t*), *s*(*t*) and *h*(*t*) which represent model trends, and the model error $\epsilon _t$. Variable *g*(*t*) represents the non-periodic modeling trend component, *s*(*t*)—periodic modeling component, and *h*(*t*)—extreme events (or holidays) which can occur with an irregular schedule. The model error $\epsilon _t$ represents the processes which are unexplained by the model. All the model variables and the error depend on time *t*. The model (3) can also incorporate a covariate i.e. an independent predictor for *y*(*t*)^47^. Therefore, the number of individuals in the population of Belarus who were 65 years or older was considered as such covariate in the model fitting process.

The autoregressive integrated moving average (ARIMA) model is implemented in R package forecast^46^. The ARIMA model is determined by the set of three parameters (*p*, *d*, *q*). The parameter *p* determines the lag of the model i.e. the number of previous observations $Y(t-1), Y(t-2), \dots , Y(t-p)$ which are used to fit the current observation *Y*(*t*). The parameter *d* stands for the “degree” of the model, i.e. for how many times the previous response has to be subtracted to ensure the required stationarity model assumption^48^. For example, for $d=0$ the newly defined response variable is $y(t) = Y(t)$, for $d=1$ the modeled variable is $y(t) = Y(t) - Y(t-1)$, and for $d=2$ the modeled variable is $y(t) = \Big [ Y(t) - Y(t-1) \Big ] - \Big [ Y(t-1) - Y(t-2) \Big ]$. The third parameter *q* stands for the number of moving average parameters within the model. The formal ARIMA modeling equation has the form:

4 $$\begin{aligned} y(t) = \mu + \sum _{i=1}^{p} \varphi _i\, y(t-i) + \sum _{j=1}^{q} \theta _j\, \epsilon _{t-j} + \epsilon _{t}. \end{aligned}$$

The formula (4) contains the response model variable *y*(*t*) which represents mortality counts (or search trend values) and is parameterized by the overall mean parameter $\mu$, the autoregressive parameters $\varphi _i$ where $i=1,2,\dots , p$, the moving average parameters $\theta _j$ and the model errors $\epsilon _{t-j}$ where $j=0,1,2,\dots , q$.

### Score computations

The non-parametric *P*-scores for mortality data for January–June of 2020 were computed based on the monthly averages $\bar{x}(t_i)$ for the first six months of the year i.e. for $t_1, t_2, \dots , t_6$. The averages were based on *five* (2015-2019) and *nine* (2011-2019) years of data. The analogous computations of *P*-scores were preformed for three Google Trends based on *five* (2015-2019) years of data.

The parametric $\mathscr {P}$-scores for mortality data were computed based on the predicted averages $\hat{\mu }(t_i)$ that came from the Prophet forecasting model and the ARIMA model. The both models were fit to two time series with *five* (January 2015–February 2020) and *nine* (January 2011–February 2020) years of monthly mortality. For all fits the *four* following epidemic months (March 2020–June 2020) were predicted by those models. The Prophet and ARIMA models were fitted *both* without a covariate and with a covariate which was the number of individuals of age 65 or older. For the Prophet model the default package settings were used while for the ARIMA model auto.arima function was used for *nine* years to determine (*p*, *d*, *q*) parameters. The corresponding ARIMA settings were used for *five* years fits since those auto.arima options were deemed to be the most reliable.

For Prophet model the $95\%$ upper bound of model fits (and forecasts for the last four months) were used to produce the expected mortality $\hat{\mu }(t_i)$ and the corresponding $\mathscr {P}(t_i)$-scores (2). Those estimates are conservative since they use the upper bound of forecasted expected mortality thus producing the lower bound estimate of $\mathscr {P}(t_i)$-scores. The forecast package only provides ARIMA upper uncertainty bounds for the model forecasts and not for the model fits. Therefore, instead of 95% upper bound those model-produced values $\hat{\mu }(t_i)$ *both* for the fitted and for the four forecasted months were used to produce the $\mathscr {P}(t_i)$-scores.

The analogous scores (2) based on *five* (January 2015–February 2020) years of monthly trends data and the Prophet model were used the produce the expected averages $\hat{\mu }(t_i)$ and the corresponding $\mathscr {P}(t_i)$-scores of three Google Trends. Only the Prophet model was fitted for Google Trends since the results for the Prophet and ARIMA models for mortality were very similar.

### Mortality and google trends data linkage

To study the associations and (potential) relationships between mortality and Google Trends the corresponding time series were compared pairwise. The comparisons were performed for the time period when they all were available, i.e. for monthly records from January 2015 to June 2020. For easier visual comparison, the series were standardized to be on the same scale. More specifically, the values of each time series were divided by the corresponding median of that series values and the resulting values were multiplied by 100. In addition to that all four standardized series were smoothed using loess function in R with span = 0.25 parameter to remove potential noise. The resulting standardized and smoothed standardized values were compared using the Granger dependency test for time series *with dependent* records^49^. The graphical summaries were also produced.

## Results

### Raw data summaries

**Figure 1:**
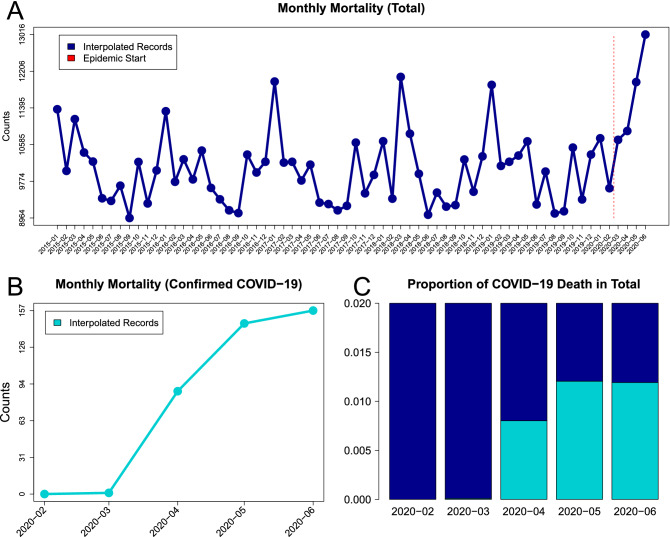
The visual summaries of the raw data for overall and COVID-19-related mortality: (**A**) Total monthly mortality counts joined by a linear interpolator. The start of the epidemic is marked with a vertical red bar. (**B**) COVID-19-related monthly mortality counts joined by a linear interpolator. (**C**) Monthly proportions of COVID-19-related mortality among the total mortality.

The visual summaries of overall and COVID-19-related mortalities are presented in Fig. 1. The overall mortality increased since April 2020 (Fig. 1 Panel A) while the number (Fig. 1 Panel B) and the proportion of COVID-19-related deaths among the total mortality remained notoriously low for the first four available months of the pandemic (Fig. 1 Panel C). In particular, the reported COVID-19-related mortality constitutes less than $1.5\%$ of the overall mortality during the reported April-June of 2020 (Fig. 1 Panel C).

**Figure 2:**
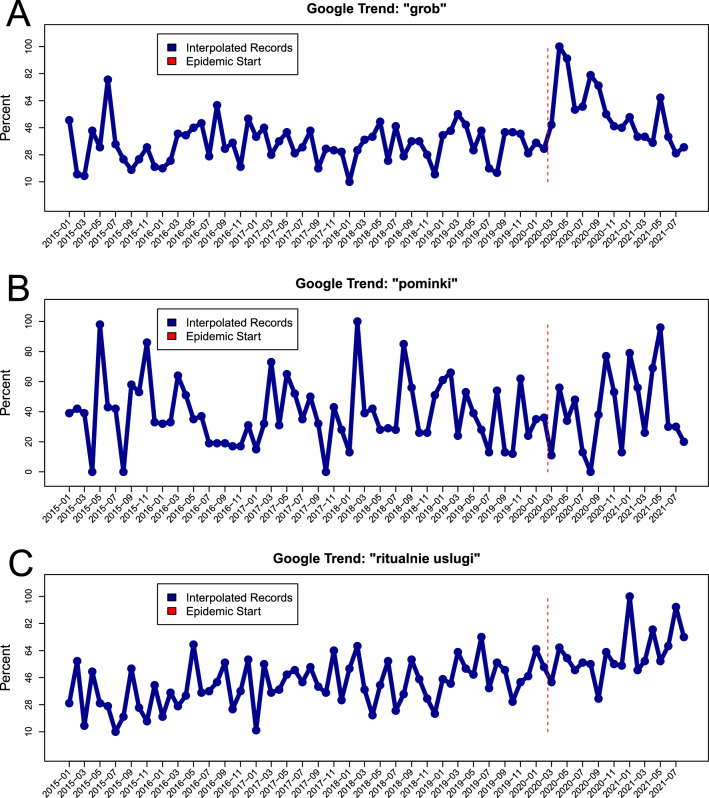
The visual summaries of the monthly raw Google trends data from January 2015 until August 2021 for (**A**) “grob” i.e. coffin, (**B**) ”pominki” i.e. memorial service and (**C**) “ritualnie uslugi” i.e. funeral services. The vertical red bar emphasizes the epidemic start, i.e. time of the first confirmed cases.

The Google Trends data visual summaries for “grob” which means coffin, ”pominki” which means memorial service and “ritualnie uslugi” which means funeral services key phrases are provided in Fig. 2. Such data are extremely variable and the trend depends heavily on the chosen key phrase. Still, the patterns do change visually after the beginning of the pandemic in March 2021. The coffin (“grob”) variable has the most pronounced trend.

### Non-parametric *P*-scores

**Figure 3:**
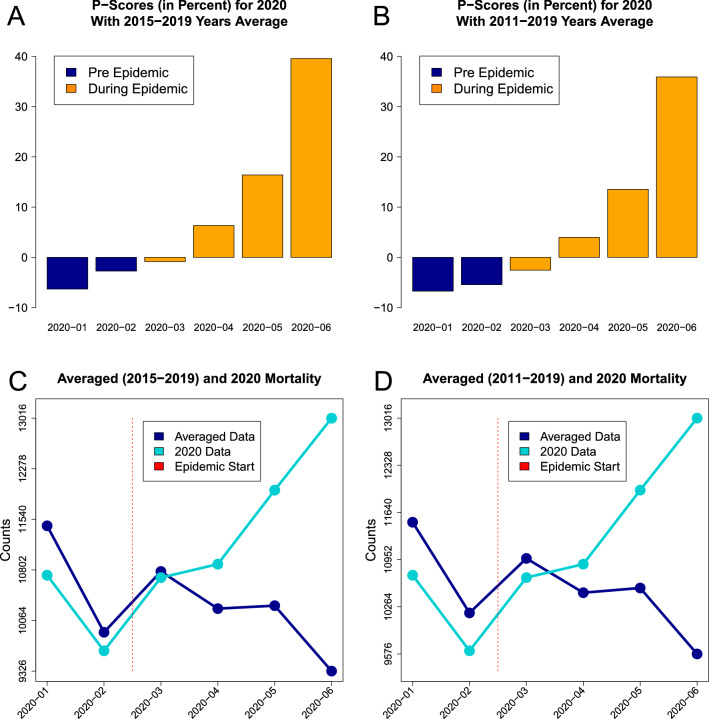
The visual summaries of non-parametric mortality *P*-scores presented for *five* (panel **A**) and *nine* (panel **B**) years where orange bars represent the epidemic period. The corresponding averaged data (dark blue) based on the previous *five* (panel **C**) and *nine* (panel **D**) years are presented along with the reported data for 2020 (cyan). Vertical red bars indicate the epidemic start period.

The non-parametric *P*-scores for mortality data based on *five* and *nine* years histories are presented in Fig. 3. The *P*-score values for early 2020 are *negative* indicating low recorded mortality. The scores *increased* from April 2020 and for the latest reported month June they were $39.56\%$ for *five* and $35.92\%$ for *nine* years histories. The summaries for *five* years averages (Fig. 3 panels A and C) and for *nine* years averages (Fig. 3 panels B and D) have very similar results while there is a clear trend of substantial incidence increase during the pandemic. The summaries from non-parametric mortality *P*-scores from Fig. 3 agree with high excess mortality identified earlier^25^.

**Figure 4:**
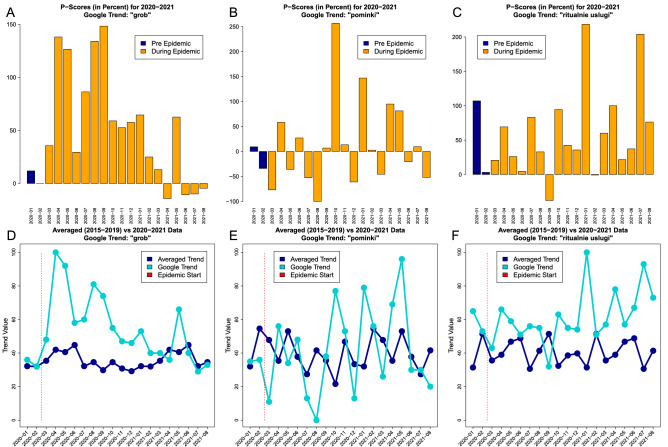
The visual summaries of non-parametric Google Trends searches *P*-scores are presented for coffin (panel **A**), memorial service (panel **B**) and funeral services (panel **C**) where orange bars are used for the epidemic period. The corresponding averaged data based on *five* years (dark blue) are plotted together with the queries data (cyan) for coffin (panel **D**), memorial service (panel **E**) and funeral services (panel **F**). Vertical red bars indicate the epidemic start period.

The non-parametric *P*-scores for the Google Trends search data based on *five* years histories are presented in Fig. 4. The Google Trends search results do vary substantially from one key phrase to the other. The key phrase “grob” i.e. coffin had *P*-score values of $138.1\%$ for April of 2020 and of $148.3\%$ for September of 2020. The peak values for coffin were observed in the middle of 2020 and later they started to decrease. The key phrase ”pominki” i.e. memorial service had a large variability with no clear trend. The largest *P*-score value was $256.48\%$ for October of 2020 and the smallest value was $-100\%$ for August of 2020. Finally, the key phrase “ritualnie uslugi” i.e. funeral services had the pronounced excess of $218.47\%$ for January of 2021. In summary: (1) the excess of non-parametric *P*-scores was observed during the pandemic period and (2) the excess was not uniform across different Google Trends, which emphasizes potential self-reporting search biases.

The presented non-parametric *P*-scores are *robust* for misspecifications since they do not depend on any model assumptions but they also cannot include covariates.

### Parametric $\mathscr {P}$-scores

**Figure 5:**
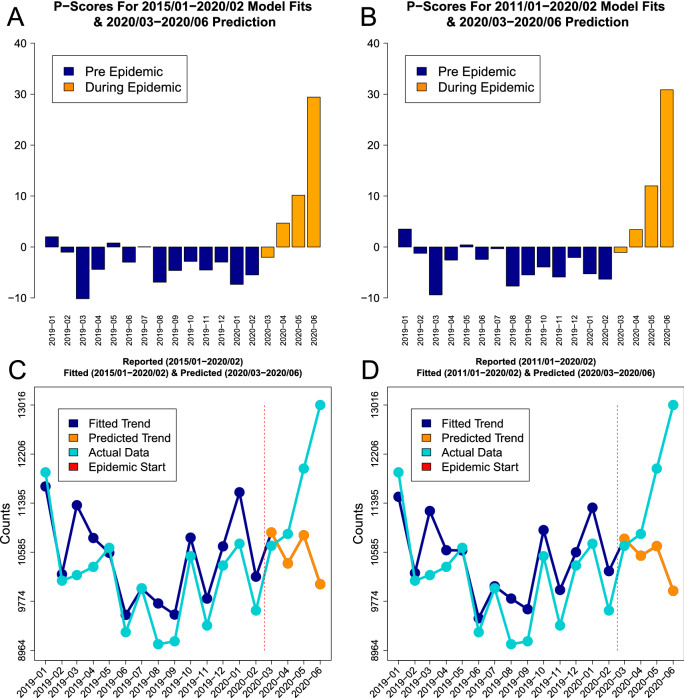
The visual summaries of parametric mortality $\mathscr {P}$-scores based on the Prophet model *without* demographic characteristics are presented for *five* (panel **A**) and *nine* (panel **B**). The orange bars represent the epidemic period. The corresponding Prophet model predictions (dark blue) based on the previous *five* (panel **C**) and *nine* (panel **D**) years are presented along the reported data (cyan). Vertical red bars indicate the epidemic start period.

The parametric $\mathscr {P}$-scores for mortality data based on the Prophet forecasting model fitted to *five* and *nine* years histories *without* demographic characteristics are presented in Fig. 5. The $\mathscr {P}$-score values for early 2020 are *negative* indicating low recorded mortality. The scores *increased* from April 2020 and for the latest reported month June they were $29.81\%$ for *five* and $30.89\%$ for *nine* years histories. The summaries for *five* years histories (Fig. 5 panels A and C) and for *nine* years histories (Fig. 5 panels B and D) have very similar results while there is a clear trend and substantial mortality increase during the pandemic.

The corresponding version of the Prophet model *with* demographic characteristics (i.e. the number of individuals of age 65 and older) produced very similar results ($33.29\%$ and $33.13\%$) which have been summarized in Supplement Fig. S1. In addition to that, the comparisons of the Prophet model fitting for *nine* years *with* and *without* demographic characteristics are presented in Supplement Fig. S2. There is no indication from such comparisons that the ageing population had an effect on mortality during the past pre-pandemic years while the pandemic period was substantially different from pre-pandemic period.

The parametric $\mathscr {P}$-scores for mortality data based on the ARIMA model had very similar trends and results to the ones from the Prophet model. This was true *both* for the models *with* demographic characteristics (i.e. the number of individuals of age 65 and older) and for those *without* them. The scores *increased* from April 2020 and for all ARIMA models. For the models *without* demographic characteristics for the latest reported month June the scores were $31.07\%$ for *five* and $33.43\%$ for *nine* years histories. For the models *with* demographic characteristics (i.e. the number of individuals of age 65 and older) for the latest reported month June the scores were $29.46\%$ for *five* and $35.59\%$ for *nine* years histories. The corresponding visual summaries are provided in Supplement Fig. S3 and Fig. S4. In addition to that, the comparisons of the ARIMA model fitting for *nine* years *with* and *without* demographic characteristics are presented in Supplement Fig. S5. In the same way as for the Prophet model there is no indication from such comparisons that the ageing population had an effect on mortality over the past pre-pandemic years while the pandemic period was substantially different from pre-pandemic period.

**Figure 6:**
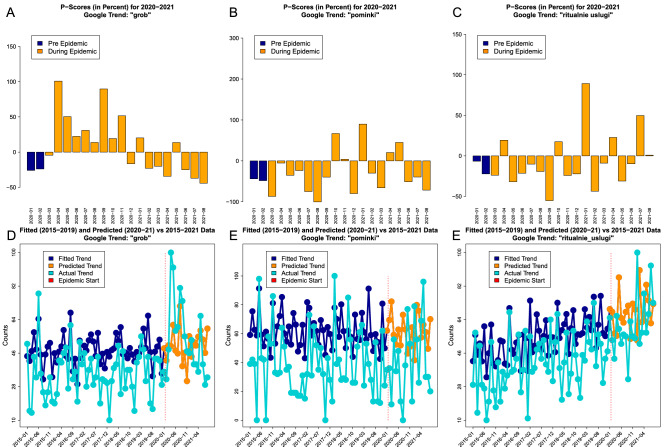
The visual summaries of parametric Google Trends searches $\mathscr {P}$-scores are presented for the coffin (panel **A**), memorial service (panel **B**) and funeral services (panel **C**) where orange bars are used for the epidemic period. The corresponding model predictions based on *five* years (dark blue) are plotted together with the queries data (cyan) for coffin (panel **D**), memorial service (panel **E**) and funeral services (panel **E**). Vertical red bars indicate the epidemic start period.

The parametric $\mathscr {P}$-scores for the Google Trends search data based on the Prophet forecasting model are presented in Fig. 6. In the same way as for non-parametric scores the results for $\mathscr {P}$-scores do vary substantially from one key phrase to the other. The key phrase “grob” i.e. coffin had the pronounced excess of $97.1\%$ for April of 2020 and $86.6\%$ for September of 2020. The peak values for coffin were observed in the middle of 2020. The key phrase ”pominki” i.e. memorial service did not have a clear trend over the study period and large variability was observed. The largest score was the excess of $66.91\%$ for October of 2020 and the smallest score was the shortage of $-100\%$ for August of 2020. Finally, the key phrase “ritualnie uslugi” i.e. funeral services had the pronounced excess of $95.40\%$ for January of 2021. In summary: (1) the excess of parametric $\mathscr {P}$-scores was observed during the pandemic period, (2) the excess was not uniform across different Google Trends which emphasizes potential self-reporting search biases, and (3) the parametric scores were more “smooth” and “less extreme” than the non-parametric scorers causing smaller variability and less extreme values.

### Compassion of excess mortality scores across methods and regions

The formal comparison between non-parametric (1) and parametric (2) scores and the corresponding ”excess mortality“ based on *five* years histories are presented in Tables 1 and 2. The summaries for *nine* years histories are very similar and are available in Supplement Tables S1 and S2. Regardless of the method both *P* and $\mathscr {P}$ scores increased since March 2020 and were in the range from $\sim 10\%$ to $\sim 20\%$ for May 2020 and from $\sim 29\%$ to $\sim 40\%$ for June 2020. The corresponding excess mortality estimates were from 1116 to 2035 deaths for May 2020 and from 2953 to 3690 deaths for June 2020. For all models the estimated “excess death” far exceeded the reported COVID-19-related mortality for the same time period (Fig. 1).

**Table 1:** The values of *P* and $\mathscr {P}$ for the first six months of 2020 based on *five* previous years of historic data for different modeling approaches.

Method	2020-01	2020-02	2020-03	2020-04	2020-05	2020-06
Non-parametric	− 6.30	− 2.74	− 0.83	6.33	16.40	39.56
Prophet	− 7.23	− 5.08	− 1.82	4.40	10.29	29.35
Prophet & 65+	− 5.47	− 2.94	0.65	7.86	13.37	33.29
ARIMA	7.11	− 4.49	8.16	9.67	20.49	31.07
ARIMA & 65+	4.45	− 3.51	6.88	7.84	19.33	29.46

**Table 2:** The estimated excess mortality counts for the first six months of 2020 based on *five* previous years of historic data for different modeling approaches. Negative values indicate less than predicted counts.

Method	2020-01	2020-02	2020-03	2020-04	2020-05	2020-06
Non-parametric	− 721	− 271	− 90	648	1686	3690
Prophet	− 836	− 515	− 198	459	1116	2953
Prophet & 65+	− 621	− 291	69	793	1411	3251
ARIMA	712	− 452	806	960	2035	3086
ARIMA & 65+	457	− 350	688	791	1938	2962

In the meantime, the excess mortality is expected everywhere during the pandemic. Therefore, since *P* and $\mathscr {P}$-scores have similar values the formal comparisons of Belarusian *P*-scores with neighboring and more distant countries can provide a formal comparison of the pandemic burden. In particular, based on the interactive maps of ourworldindata.org^50^ all scores computed for Belarus for June 2020 were higher than for *all* neighboring counties with $5\%$ for Poland, $5\%$ for Ukraine, $8\%$ for Russia, $11\%$ for Lithuania, $11\%$ for Latvia. Our findings also agree with exceptionally high mortality in Belarus which has been documented earlier^25^ but provide extra insides in terms of multiple estimation approaches (non-parametric, Prophet and ARIMA) with the inclusion of demographic characteristics (i.e. number of individuals older than 65) in the study.

As of November 2021 Belarus is *the only* country among its neighbors where no mortality has been reported past June 2020. Moreover, based on the available data, the mortality demonstrated the exponential growth from March 2020 until the reporting ended. It is also worth mentioning that June *was not* the peak month for mortality in the region. For example, neighboring Poland has demonstrated the peak in excess mortality in November 2020 with the score value of $117\%$. The other available studies of excess mortality also indicate the highest underreporting COVID-19 rate for Belarus in comparison to all neighbors^51^ which agrees with the findings of this manuscript.

For a complete picture, the analysis results for Belarus were compared with the more distant Western European neighbors: United Kingdom, Spain, Belgium, Italy, Netherlands, and France^52^. For those countries the corresponding *P*-scores for peak weeks in April 2020 were 105–108% for the United Kingdom, 117–156% for Spain, 90–105% for Belgium, 55–81% for Italy, 52–75% for Netherlands, and 57–65% for France depending on the week^53^. In the meantime, the direct comparison of Western European pandemic “peak” in April with the values in June for Eastern Europe is not correct: the epidemic “peak” in Western Europe was exactly in April, which was followed by a steady decline while in Eastern Europe and in Belarus in particular the epidemic was just starting in April and peaked much later than June. The manuscript findings also agree with the previous study which concluded that early lockdowns reduced mortality^54^.

### Mortality and google trends data relationships

**Figure 7:**
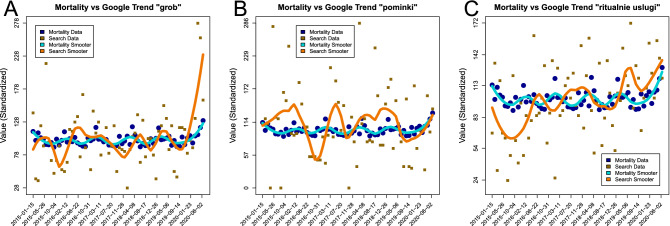
The visualization of pairwise comparisons between the standardized mortality records and the standardized Google Trends for (**A**) ”grob” i.e. coffin, (**B**) ”pominki” i.e. memorial service and (**C**) “ritualnie uslugi” i.e. funeral services key phrases. The standardized mortality values are presented as blue dots, while the standardized search data are presented as brown squares. The corresponding smoothers are presented as cyan and dark orange lines.

The pairwise summaries of the standardized and smoothed standardized time series are presented in Fig. 7. The smoothed trends appear visually related, which is especially pronounced for ”grob” i.e. coffin and for “ritualnie uslugi” i.e. funeral services variables.

The more formal comparisons of Granger’s test *p*-values for scaled ($p_{GR}$) and smoothed scaled ($p_{GR(Sm)}$) trends are provided in Table 3.

**Table 3:** The summary of *p*-values for standardized (*GR*) and smoothed standardized (*GR*(*Sm*)) data from Granger’s test.

Name	$p_{GR}$	$p_{GR(Sm)}$
”grob” (coffin)	0.14	$<0.01$
”pominki” (memorial service)	0.08	$<0.01$
“ritualnie uslugi” (funeral services)	0.07	0.08

The results of Granger’s test indicate moderate significance for scaled and strong significance for smoothed scaled data.

## Discussion

The findings of this study suggest that there are indications that an approach with limited NPIs could have resulted in the overall mortality increase. While such an increase was observed among all neighboring countries where more stringent NPIs were implemented, the Belarusian mortality was the highest for the last reported month of June 2020. The exact causes of such increase in the overall mortality are unknown, but the timeline of the increase corresponded to the pandemic time period in Belarus. Either way, there are indications that the overall mortality in Belarus during the pandemic period which was reported was substantially higher than expected otherwise and also higher than for all neighboring countries.

The limited NPIs were the primary factors that distinguished Belarus from its direct neighbors. The direct comparisons of the county’s NPIs and the mortality rates, however, did not necessarily imply causal relationship. There could be many other factors, which affected the increase in mortality, such as multiple differences in healthcare systems and population structure between the countries. In particular, the effects of the population age structure changes were investigated. Based on the available demographics data there were (1) no substantial changes in the number of individuals in the age group of 65+ during the studied period and (2) the inclusion of such data into the models resulted in similar mortality predictions and scores as from models without such data.

The inclusion of Google Trends search data and the corresponding analysis, served as an auxiliary indicator that a higher increase in mortality could have happened until the end of 2020. No further studies, however, can be performed on exact mortality counts until more data will become available.

The retrospective analysis of mortality for a single region and the comparisons of such analyses between regions remain challenging due to inherited limitations. At a first glance, the analysis of excess mortality seems to be a perfect measure of the epidemic burden. Moreover, the use of standardized scores for comparison between regions or countries is handy. In reality, the mortality data accumulation and reporting are far from perfect and have flaws^55^. For Belarus those limitations are exacerbated by limited reporting which does not cover any time past June 2020.

The first challenge is the frequency and completeness of reporting and the corresponding reporting lag. In particular, while the lab confirmed COVID-19 mortality during the pandemic is reported very frequently (e.g. daily), the overall mortality is reported with some delay and for longer time periods i.e. it can be reported weekly, monthly, quarterly or (even) annually. Moreover, the overall mortality tends to be incomplete for developing countries and even for developed countries the proper and complete documentation of *all cases* can take up to one year and beyond^40^. This happens since it may not be immediately possible to identify causes of death and, therefore, it may take some time to produce the appropriate death certificates and to incorporate them into the statistics^40^. As a consequence, even though, those cases do not comprise the majority of statistics, they can introduce reporting delays.

The second challenge is the constantly changing population structure which is not always incorporated into the analysis. In particular, the non-parametric *P*-scores which are commonly used in research cannot incorporate the number of people older than 65 at each given point of time, which is critical for ageing populations like Belarus. Such analysis can only be incorporated in parametric $\mathscr {P}$-scores (2) and via modeling of means $\mu (t)$ which can include the modeling of demographic characteristics over time. This latter parametric approach also comes with limitations, since the population age structure and corresponding population sizes and total counts may not be available with the studied time intervals precision. Therefore, averages over time for longer periods have to be used instead. This was the case for the presented Belarusian analysis where the population size and age structure are only reported annually.

The third challenge is that various demographic and social processes are happening differently within the society during the pandemic. In particular, the overall mortality is affected by multiple factors which can *both* increase or decrease the mortality. Therefore the excess mortality does not directly reflect the mortality caused by COVID-19. For example, individuals can die in excess numbers during the epidemic due to *other causes* unrelated to SARS-CoV-2, such as anxiety and related health complications (e.g. stroke), suicides or inability of the healthcare system to accommodate planned hospitalizations and surgeries at that time. At the same time epidemics may also have a decreasing effect on the overall mortality, since people may travel less and the number of travel-related accidents and deaths can decrease^55^. Therefore, the overall mortality in our study should be interpreted as the *overall COVID-19 burden* on the society as a whole rather than the COVID-19 induced death. Moreover, the exact (clinical) causes of each death are not reported for Belarus. As a consequence, the mortality summaries by clinical cause cannot be directly compared or attributed to COVID-19.

The fourth challenge comes from the study of the Google Trends search data^56^. We had to refer to them to *complement* the limited knowledge and to study the *alternative* sources of information about mortality during the pandemic. As a result, such analyses indeed provided us with evidence about the increased funeral-related search trends during the pandemic. Such data, however, are prone to severe reporting biases since the number of Google users changes constantly. In particular, people may constantly change frequency of use or use multiple search engines at the same time. The other limitation comes from variability of search options i.e. the list of potential key phrases can be endless. We have only focused on the three most obvious key phrases and even those had quite different time series. As a result, the Google Trends search data *are not complete* by any means and the results are *expected* to be biased in one way or the other. Still, from all studied trends the excess searches have been observed during the pandemic which were more or less pronounced.

## Supplementary Information

Supplementary Information.

## Data Availability

The analysis source code in R has been made publicly available on GitHub and can be downloaded^57^.
