# Supplementary material for: Excess mortality in Belarus during the COVID-19 pandemic as the case study of a country with limited non-pharmaceutical interventions and limited reporting
Source: Sci Rep. 2022 Mar 31;12:5475. doi: 10.1038/s41598-022-09345-z (PMC8970068; doi:10.1038/s41598-022-09345-z)
Supplement: Supplementary file 1 — Supplementary Information. [file 41598_2022_9345_MOESM1_ESM.pdf]

Excess mortality in Belarus during the COVID-19 pandemic as the case study of a country with limited non-pharmaceutical interventions and limited reporting.

Alexander Kirpich[1,*], Aleksandr Shishkin[1], Thomas A. Weppelmann[2], Alexander Perez Tchernov[3], Pavel Skums[4], and Yuriy Gankin[5]

[1]Department of Population Health Sciences, School of Public Health, Georgia State University, Atlanta, Georgia, United States of America
[2]Department of Internal Medicine, University of South Florida, Tampa, Florida, United States of America
[3]Faculty of Mechanics and Mathematics, Belarusian State University, Minsk, Belarus
[4]Department of Computer Science, Georgia State University, Atlanta, Georgia, United States of America
[5]Quantori, Cambridge, Massachusetts, United States of America
[*]akirpich@gsu.edu

ABSTRACT

Public health intervention to contain the ongoing COVID-19 pandemic significantly differed by country since the SARS-CoV-2 spread varied regionally in time and in scale. Since vaccinations were not available until the end of 2020 non-pharmaceutical interventions (NPIs) remained the only strategies to mitigate the pandemic spread at that time. Belarus in Europe is one of a few countries with high Human Development Index where no lockdowns have ever been implemented and only limited NPIs have taken place for a period of time. Therefore, the Belarusian case was evaluated and compared in terms of the mortality burden. Since the COVID-19 mortality was low, the excess *overall* mortality was studied for Belarus. Since no overall mortality data have been reported past June 2020 the analysis was complemented by the study of Google Trends funeral-related search queries up until August 2021. Depending on the model the Belarusian mortality for June of 2020 was 29% to 39% higher than otherwise expected with the corresponding estimated excess death was from 2953 to 3690 while the reported COVID-19 mortality for June 2020 was only 157 cases. The Belarusian excess mortality for June 2020 was higher than for all neighboring countries with excess of 5% for Poland, 5% for Ukraine, 8% for Russia, 11% for Lithuania and 11% for Latvia. The relationship between Google Trends and mortality time series was studied using Granger's test and the results were statistically significant. The results for Google Trends searches did vary by key phrase with the largest excess of 138% for April 2020 and 148% for September 2020 was observed for a key phrase "coffin", while the largest excess of 218% for January 2021 was observed for "funeral services". In summary, there are indications of the excess overall mortality in Belarus, which is larger than the reported COVID-19-related mortality.

Supplemental Material

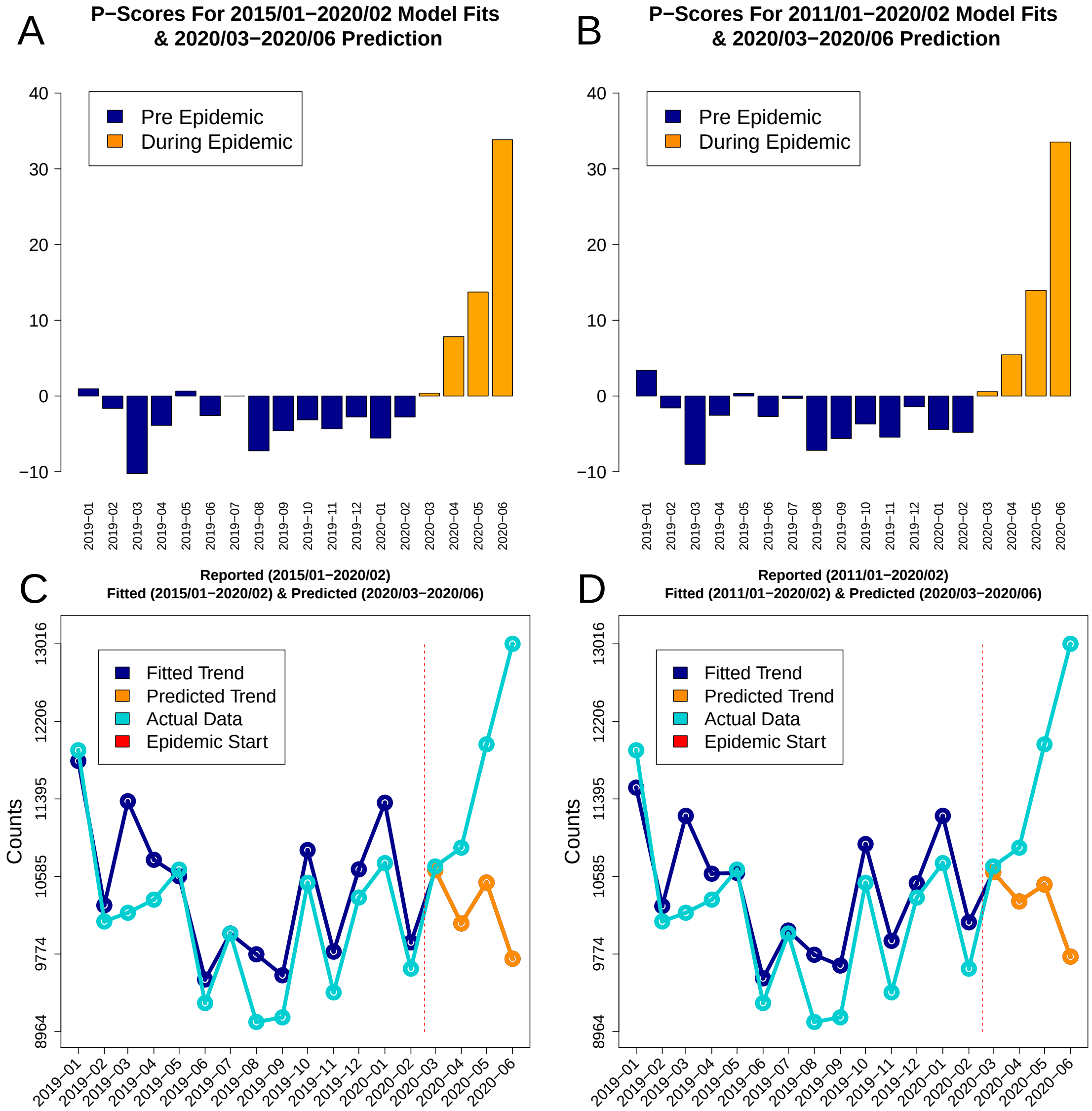

Figure S1. The visual summaries of parametric mortality $\mathscr{P}$-scores based on the Prophet model *with* demographic characteristics (i.e. with 65+ covariate) are presented for *five* (panel A) and *nine* (panel B). The orange bars represent the epidemic period. The corresponding Prophet model predictions (dark blue) based on the previous *five* (panel C) and *nine* (panel D) years are presented along the reported data (cyan). Vertical red bars indicate the epidemic start period.

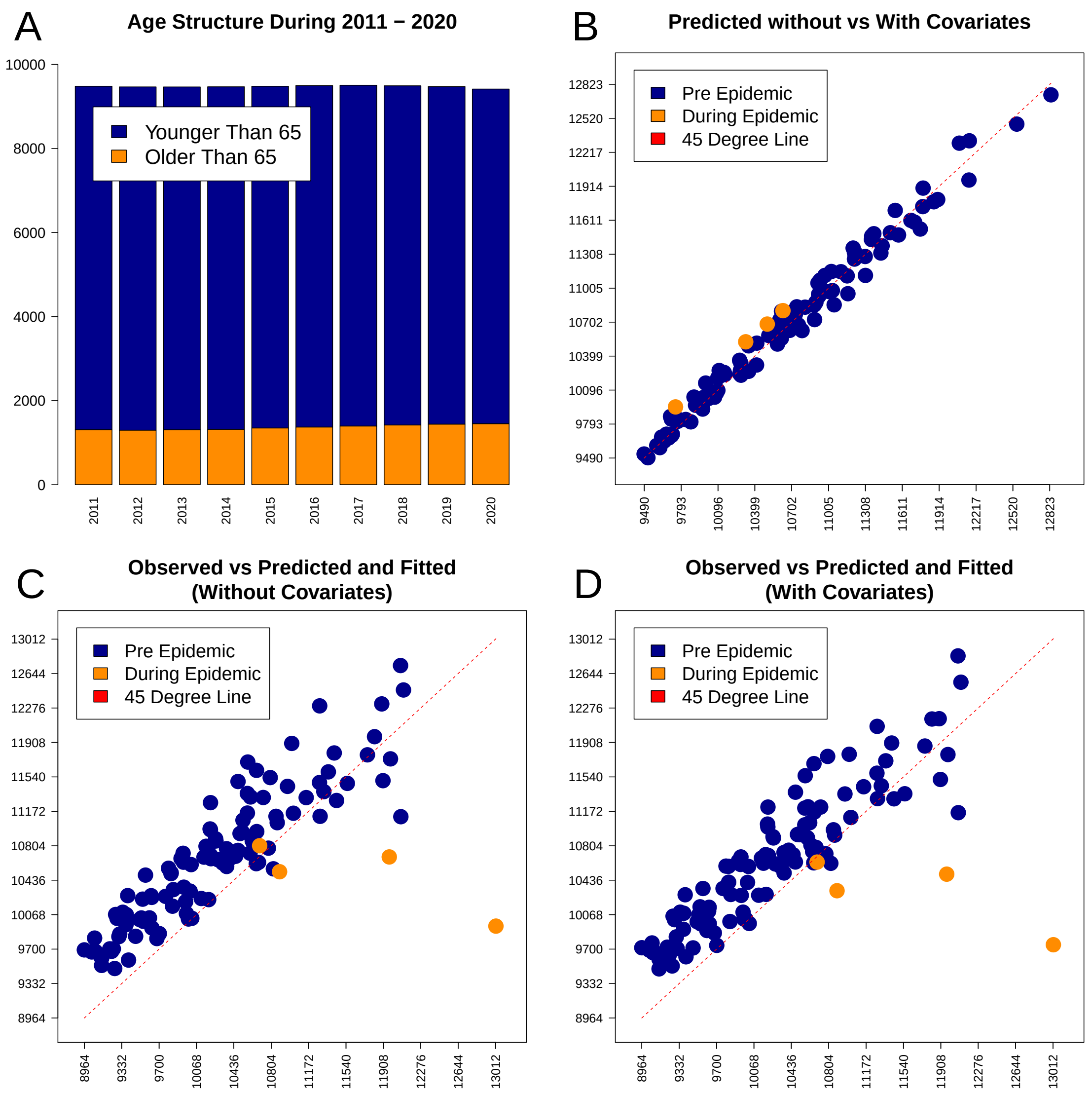

Figure S2. The visual summaries of the Prophet model fits compared without and with a 65+ covariate for nine year histories: A) The total population and the proportion of individuals of age 65+. B) Comparison of predictions for two models: with vs without 65+ covariate. Summaries of observed vs predicted for for model without (C) and with (D) covariate.

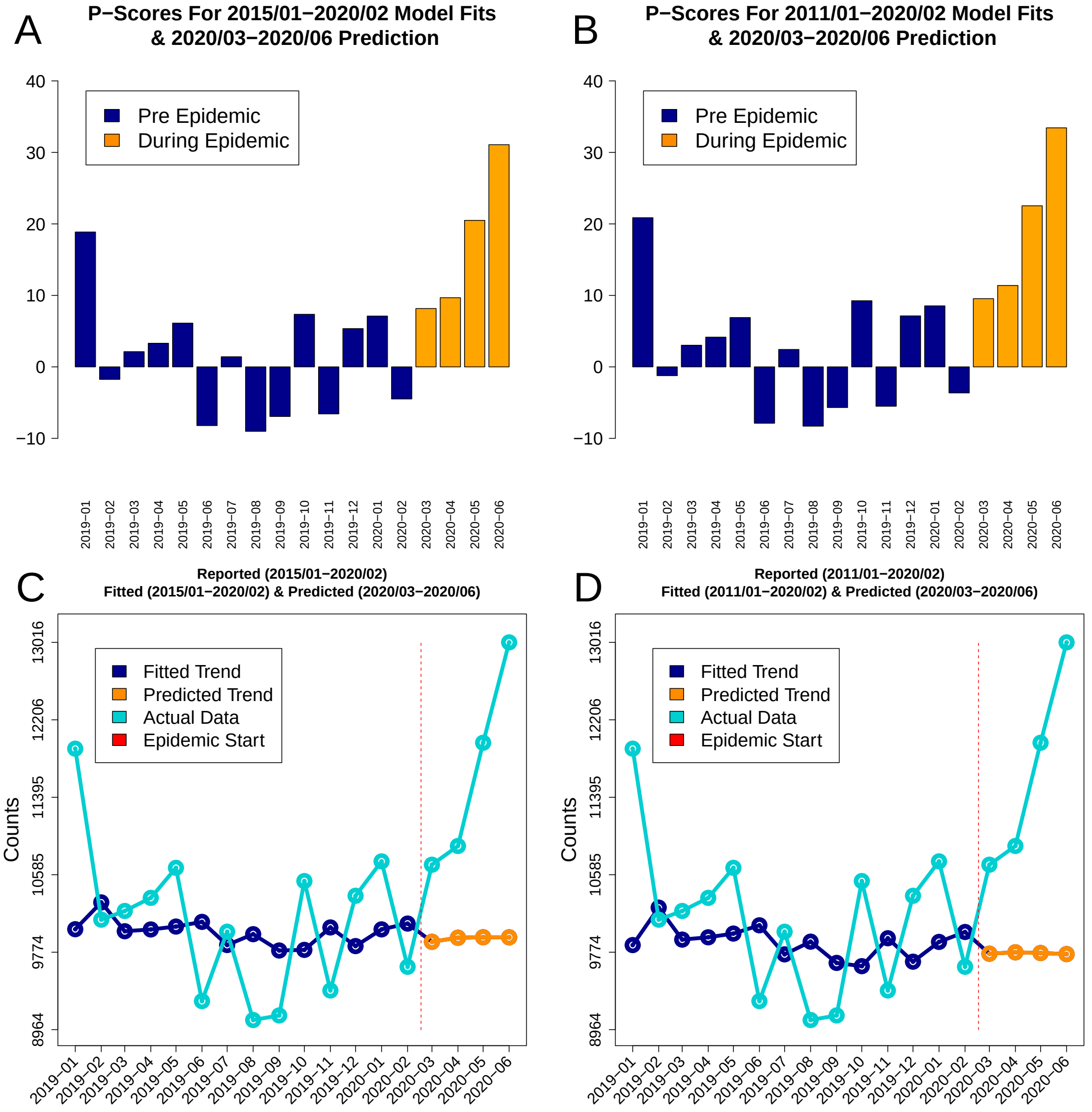

Figure S3. The visual summaries of parametric mortality $\mathscr{P}$-scores based on the ARIMA model *without* demographic characteristics are presented for *five* (panel A) and *nine* (panel B). The orange bars represent the epidemic period. The corresponding ARIMA model predictions (dark blue) based on the previous *five* (panel C) and *nine* (panel D) years are presented along the reported data (cyan). Vertical red bars indicate the epidemic start period.

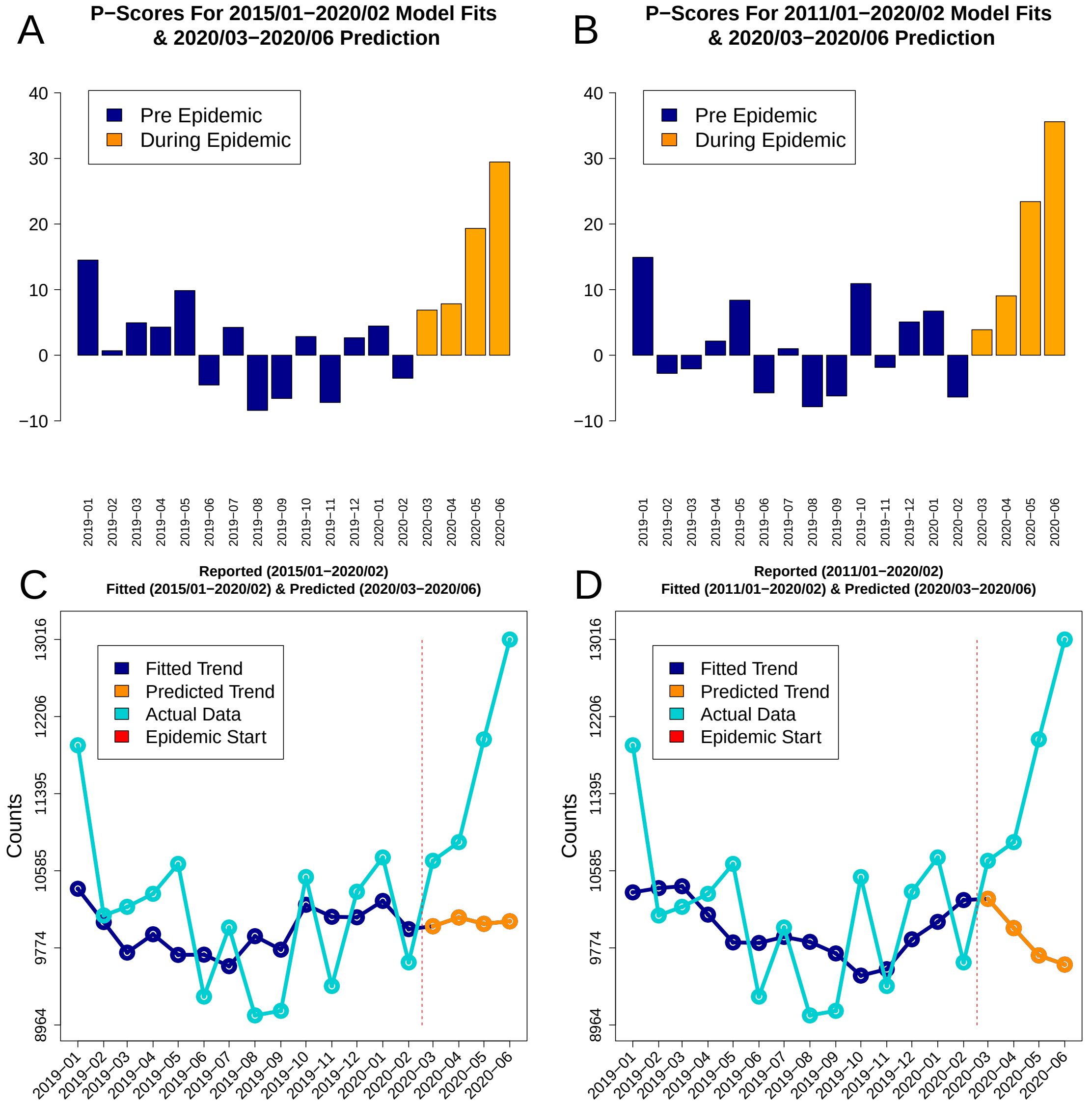

Figure S4. The visual summaries of parametric mortality $\mathscr{P}$-scores based on the ARIMA model *with* demographic characteristics (i.e. with 65+ covariate) are presented for *five* (panel A) and *nine* (panel B). The orange bars represent the epidemic period. The corresponding ARIMA model predictions (dark blue) based on the previous *five* (panel C) and *nine* (panel D) years are presented along the reported data for (cyan). Vertical red bars indicate the epidemic start period.

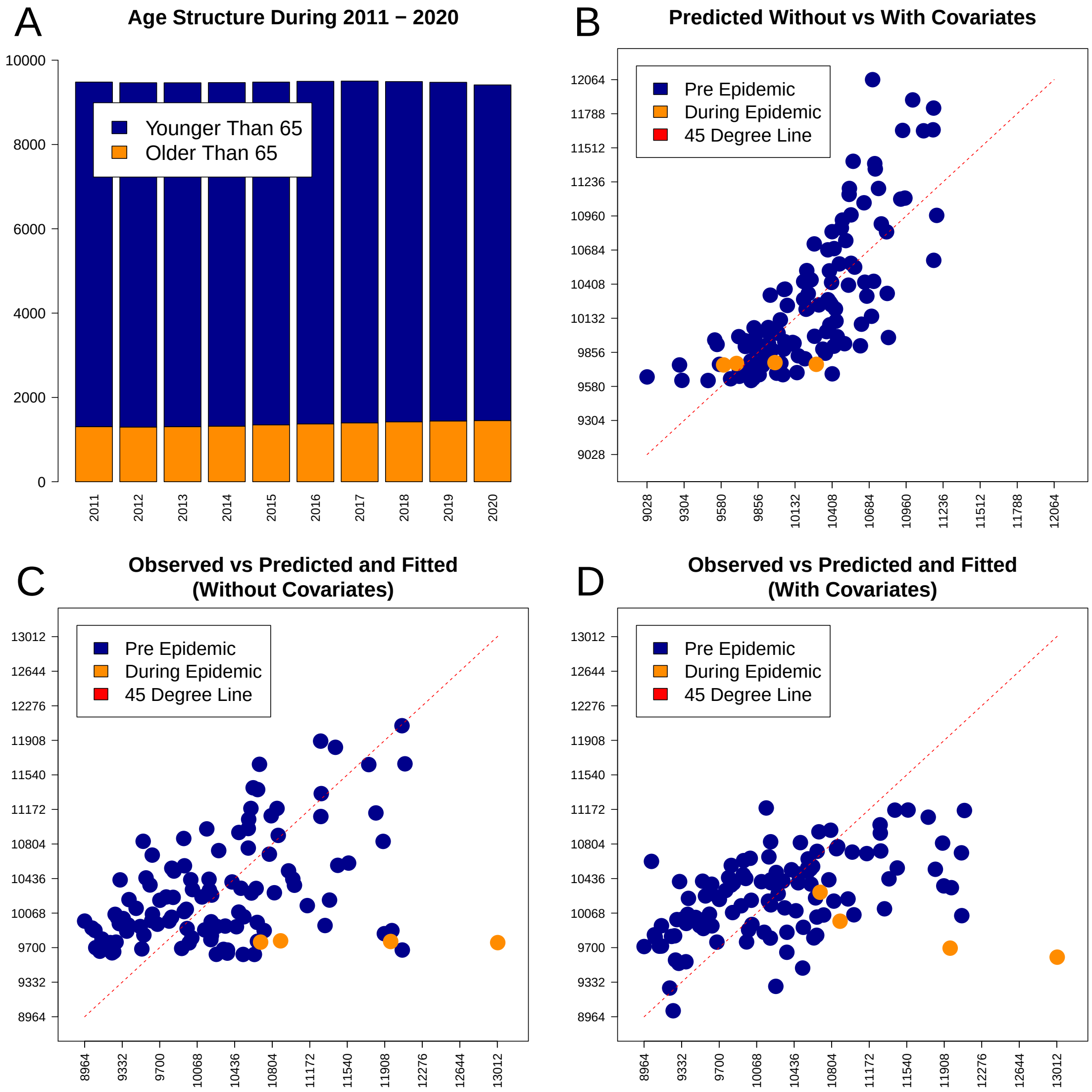

Figure S5. The visual summaries of the ARIMA model fits compared without and with a 65+ covariate for nine year histories: A) The total population and the proportion of individuals of age 65+. B) Comparison of predictions for two models: with vs without 65+ covariate. Summaries of observed vs predicted for for model without (C) and with (D) covariate.

Method	2020-01	2020-02	2020-03	2020-04	2020-05	2020-06
Non-parametric	-6.73	-5.43	-2.55	3.98	13.55	35.92
Prophet	-5.38	-5.81	-1.17	3.73	11.84	31.03
Prophet & 65+	-4.49	-4.71	0.38	5.16	13.96	33.13
ARIMA	8.53	-3.65	9.54	11.38	22.53	33.43
ARIMA & 65+	6.73	-6.38	3.89	9.05	23.42	35.60

Table S1. The values of P and $\mathscr{P}$ for the first six months of 2020 based on *nine* previous years of historic data for different modeling approaches.

Method	2020-01	2020-02	2020-03	2020-04	2020-05	2020-06
Non-parametric	-773	-553	-280	416	1428	3440
Prophet	-610	-593	-126	391	1267	3082
Prophet & 65+	-504	-475	40	534	1466	3239
ARIMA	843	-364	931	1113	2200	3261
ARIMA & 65+	677	-655	400	903	2270	3417

Table S2. The estimated excess mortality counts for the first six months of 2020 based on *nine* previous years of historic data for different modeling approaches. Negative values indicate less than predicted counts.
